# "If the patients decide not to tell what can we do?"- TB/HIV counsellors' dilemma on partner notification for HIV

**DOI:** 10.1186/1472-698X-11-6

**Published:** 2011-06-03

**Authors:** Barnabas N Njozing, Kerstin E Edin, Miguel San Sebastián, Anna-Karin Hurtig

**Affiliations:** 1 St. Mary Soledad Catholic Hospital, Mankon, Bamenda, P.O.Box 157, Cameroon; 2 Department of Public Health and Clinical Medicine, Unit of Epidemiology and Global Health, Umeå University, Umeå, 901 85, Sweden; 3 Swedish Research School for Global Health, Umeå University, 901 85, Umeå, Sweden; 4 Umeå Centre for Gender Studies, Umeå University, Sweden

## Abstract

**Background:**

There is a global consensus towards universal access to human immunodeficiency virus (HIV) services consequent to the increasing availability of antiretroviral therapy. However, to benefit from these services, knowledge of one's HIV status is critical. Partner notification for HIV is an important component of HIV counselling because it is an effective strategy to prevent secondary transmission, and promote early diagnosis and prompt treatment of HIV patients' sexual partners. However, counsellors are often frustrated by the reluctance of HIV-positive patients to voluntarily notify their sexual partners. This study aimed to explore tuberculosis (TB)/HIV counsellors' perspectives regarding confidentiality and partner notification.

**Methods:**

Qualitative research interviews were conducted in the Northwest Region of Cameroon with 30 TB/HIV counsellors in 4 treatment centres, and 2 legal professionals between September and December 2009. Situational Analysis (positional map) was used for data analysis.

**Results:**

Confidentiality issues were perceived to be handled properly despite concerns about patients' reluctance to report cases of violation due to apprehension of reprisals from health care staffs. All the respondents encouraged voluntary partner notification, and held four varying positions when confronted with patients who refused to voluntarily notify their partners. Position one focused on absolute respect of patients' autonomy; position two balanced between the respect of patients' autonomy and their partners' safety; position three wished for protection of sexual partners at risk of HIV infection and legal protection for counsellors; and position four requested making HIV testing and partner notification routine processes.

**Conclusion:**

Counsellors regularly encounter ethical, legal and moral dilemmas between respecting patients' confidentiality and autonomy, and protecting patients' sexual partners at risk of HIV infection.

This reflects the complexity of partner notification and demonstrates that no single approach is optimal, but instead certain contextual factors and a combination of different approaches should be considered. Meanwhile, adopting a human rights perspective in HIV programmes will balance the interests of both patients and their partners, and ultimately enhance universal access to HIV services.

## Background

In recent years, there has been a global consensus towards the rapid scale-up and universal access to human immunodeficiency virus (HIV) services. This is especially so in sub-Saharan Africa which bears the overwhelming brunt of the epidemic. However, for this to be feasible, knowledge of one's HIV-positive status is a prerequisite. HIV testing and counselling serves this purpose since it is a critical prevention and treatment tool in the control of HIV infection [1]. The conventional client-initiated approach to HIV testing, also known as voluntary counselling and testing (VCT), has led many people to know their HIV status, reduce or modify risky behaviours, and prevent HIV transmission to others. Yet, less than 40% of the population in sub-Saharan Africa living with HIV know their status [2]. Consequently, in populations with generalised HIV epidemic (where HIV prevalence is consistently over 1% in pregnant women), provider-initiated testing and counselling (PITC), which is counselling recommended by health care providers to every person attending health care facilities has since 2007 been recommended as a supplement to VCT [3]. This approach ensures the systematic diagnosis of HIV and thereby facilitating patients' access to HIV services.

A major area of concern in counselling is how to encourage patients to disclose their HIV status after testing. Disclosure is defined literally as the action of making new or secret information known [4]. HIV disclosure however, is defined as a 'complex and multifaceted process of making a voluntary or involuntary decision about whom to inform about one's serostatus, why, when, where and how' [5]. This is particularly challenging when it comes to informing patients' sexual partners, also referred to as partner notification. The three approaches to partner notification include: i) *source referral*, whereby the health care provider encourages the patients to alert their partners themselves; ii) *provider referral*, whereby the health care provider notifies the partners with the consent of the patients while respecting the patients' confidentiality; and iii) *conditional referral*, whereby the patients in agreement with the health care provider are supposed to inform their partners within a given time frame otherwise the health care provider will do so (but without revealing the patients' identity) [6]. The increasing emphasis on partner notification in HIV control programmes is backed by empirical evidence that it is an effective strategy of preventing HIV transmission to sexual partners at risk, and also promoting early diagnosis and prompt treatment to those found infected [7,8].

The eventual motivation to notify one's sexual partners is influenced by the patients' ethical responsibility and concern for the partners' health, the desire for social support, the severity of the disease, culturally related factors [9], and the important role played by counsellors [1,10]. However, counsellors are frequently frustrated by the low rates of HIV-positive patients who actually inform their partners about their status [11,12]. These low rates of disclosure eventually lead to the likelihood of treatment default since such patients would prefer not to be traced in the community [11]. It also leads to lost opportunities for prevention of new infections in partners at risk, and inability to access appropriate HIV services for both the patients and their partners [13].

In 2001, the Cameroon government initiated a decentralised approach of its national antiretroviral treatment (ART) programme to the district level. TB and HIV services have been integrated and counselling for HIV amongst TB patients is routine and free of charge. Since May 2007, ART and drugs for treating HIV opportunistic infections have been provided without costs to eligible patients. The decentralisation of HIV/AIDS care has improved access to HIV services including partner notification, especially amongst women. It has been documented that 86.3% of women informed their main sexual partners about their HIV status. This was especially so for married compared to unmarried women (90.0% vs. 80.6%). However, only 46% of these women knew their partners' serostatus [14]. Plausible explanations for the difference between men and women are that either the male partners' status was unknown or they refused to share their results with their female partners. The results support other studies identifying gender inequality as one of the factors fuelling HIV transmission in sub-Saharan Africa, and are noteworthy because non-disclosure jeopardizes effective HIV preventive efforts [15,16].

Considering the high HIV co-infection rate amongst TB patients in the country (40.4%) [17], TB/HIV counsellors are at the frontline to ensure that co-infected patients and their families access HIV services. This study was therefore conducted to explore TB/HIV counsellors' perspectives on confidentiality and partner notification; the challenges encountered in the process; and the strategies used to address them. This will not only improve our knowledge about the complexities surrounding partner notification, especially amongst this particular group of patients with high co-infection rates, but also provide insights leading to possible strategies towards a better HIV prevention and control.

## Methods

### Study setting

Cameroon is divided into 10 regions with a population of over 18 million inhabitants. This study was conducted in the Northwest region which comprises seven administrative divisions. Bamenda is its capital, and the region has over 2.1 million inhabitants that are mostly English-speaking. In the 2004 national health and demographic survey, the national HIV prevalence was 5.4%, and the region had the highest HIV prevalence of 8.7%; 11.9% for females and 5.2% for males [18]. There are presently 13 approved HIV/AIDS treatment centres in the region [17]. Four of these were purposively selected for the study as follow-up to other studies evaluating TB/HIV collaborative activities in the region [19,20]. The selection of these centres was based on the following: i) their fairly comparable patient load, ii) their accessibility, iii) the diversity of patients treated since they serve both rural and urban populations, and iv) the similarity in the services provided since they all have CD4 machines used to monitor the immune status of HIV-positive patients. The centres are connected to faith-based hospitals (Banso Baptist Hospital, Mbingo Baptist Hospital and Njinikom Catholic Hospital) and a public hospital (Regional Hospital Bamenda).

### HIV counselling services for TB patients

All newly diagnosed TB patients in the treatment unit, plus the referrals from other health services whose HIV status is unknown are routinely offered counselling and testing for HIV using the 'opt-out' approach. Basic information about HIV and its link to TB is provided. Patients are also educated about informed consent, confidentiality of their test result, the benefits of testing including free ART, co-trimoxazole preventive therapy, and the possibility of disclosing their HIV status to relatives. For those who consent, test results are available within a few hours using rapid diagnostic test kits. Post-test counselling is offered on the same day or as the need arises to all patients regardless of the HIV status, and includes certain support services for HIV-positive persons.

### Study design and data collection

The counsellors were approached and asked about their willingness to participate and all expressly accepted. The first author performed the interviews using an inquiry guide with questions about the counsellors' background, the nature and content of counselling, and how confidentiality and partner notification issues were handled. Based on preliminary comparative analysis of 30 conducted interviews (7-8 in each of the 4 study sites), it was decided that further interviews would probably not yield much more additional information in relation to the research question [21].

From the interviews with the counsellors, legal issues emerged regarding confidentiality and partner notification for HIV. To obtain a legal perspective about these concerns, two additional interviews were conducted with a lawyer and a judge. All the interviews were carried out from September to December 2009 and were conducted in English. Each of the interviews lasted between 45- 90 minutes, were tape-recorded and transcribed verbatim by the first author. The tapes and transcripts were de-identified to ensure anonymity.

### Data analysis

**Figure 1:**
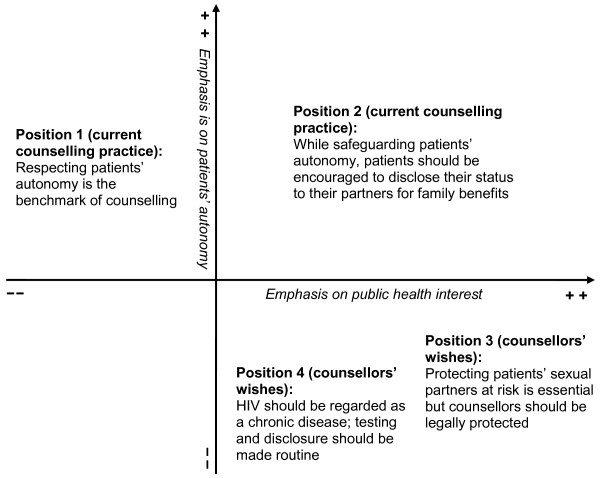
**Positional map depicting counsellors' reflections about partner notification for HIV**.

Situational Analysis [22] was used to analyse the data. The transcripts were initially read through several times to obtain a thorough understanding of the participants' views regarding confidentiality and partner notification. Thereafter, traditional Grounded Theory coding of all the texts was performed manually. The codes from the different transcripts were then reviewed while maintaining the principle of constant comparison. Codes that contained similar ideas regarding confidentiality and partner notification were grouped together. From the grouped codes, four categories were developed which represented the different positions taken by participants regarding confidentiality and partner notification. Finally, a positional map was constructed as a visual representation of these four categories (Figure 1). Two axes were used to map the positions, one with emphasis on patients' autonomy (x-axis), and the other with emphasis on public health interest (y-axis). The axes reflect the fundamental questions or debates surrounding confidentiality and partner notification for HIV.

### Trustworthiness of the study

Review of relevant literature and findings from studies in the region ensured reliability and validity of our interview material. Moreover, the first author's pre-understanding of the local context having been involved in TB/HIV management in the region built trust in the participants, and encouraged free flow of discussions. However, to be able to discover new knowledge and explore new ideas, a well prepared interview guide was used as a means of putting the contextual pre-understanding within brackets [23]. Also, to ensure credibility of the findings, many joint briefing and analysis sessions were conducted during the data collection and analysis phase with members of the research team and a senior local resource person. Feedback of the results was also provided to the relevant authorities/ethical bodies to further ensure credibility of the findings.

### Ethical approval

Ethical approval for the study was obtained from the Regional Delegation of Public Health for the Northwest Region (N°401/NWP/PDPH/08). Administrative clearance was obtained from the Internal Review Board of the Regional Hospital in Bamenda, the Cameroon Baptist Convention Health Board Institutional Review Board (IRBC20090112ez: IRB2007-09), and St. Martin de Porres Catholic Hospital Njinikom. Moreover, verbal consent was obtained from each participant after explanation of the study objectives and guarantee of secrecy.

## Results

A total of 32 participants were interviewed; 30 counsellors (24 females and 6 males, probably mirroring the high proportion of female counsellors), and 2 legal professionals. Their ages ranged from 27 to 55 years with a mean age of 36 years. A summary of the participants' characteristics is presented in Table 1. The findings are presented in two parts. Firstly, about how confidentiality issues are handled in the study settings, followed by a description of how partner notification for HIV is addressed. Secondly, a detailed description is provided regarding the various positions taken by the counsellors in relation to partner notification for HIV. Relevant quotations from the participants are provided in italics.

**Table 1:** Characteristics of interview participants (N = 32)

Characteristic	Number
**Sex**	
Male	8
Female	24
**Professional background**	
Nurse/counsellor	16
Full time counsellor	4
Community relay agent/counsellor	5
Social worker/counsellor	5
Legal professionals	2
Counsellor training*	
<1 week initial training + refresher courses	15
1-2 weeks initial training + refresher courses	10
1-2 months initial training + refresher courses	4
>2 months initial training + refresher courses	1
Working experience*	
0-2 years	4
2 - 5years	16
>5 - 10 years	10

*Excluding two legal professionals

### Dealing with confidentiality

All the participants stated that preserving confidentiality of patients' HIV result is emphasized during their training, and enforced in their counselling practice. They revealed that these measures maintain patients' trust in the health care system, facilitate HIV testing, and ultimately compliance to treatment and care. Although not all the treatment centres had clearly documented policies addressing confidentiality, the participants stated that a few cases of true breaches in patients' confidentiality had been reported to authorities and these were duly investigated and appropriate sanctions taken against the perpetrators.

*"A few patients have complained that they have heard their results in the quarters and they did not know how it got there. The authority summoned the staffs who were accused by the patients and they were later sanctioned, and one was dismissed" *(Female counsellor, 32 years old)

However, some participants mentioned that patients are often initially overwhelmed by the HIV diagnosis, and some may unintentionally disclose their status to friends and relatives but later accuse the health care providers for doing so. In addition, some mentioned that patients were generally reluctant to complain officially when they suspected that their confidentiality had been breached by staffs because they were apprehensive of reprisals. It was therefore difficult to properly investigate true cases of breach in confidentiality since such accusations from patients were only treated as rumours.

*"So far we have heard rumours but nobody has come up officially to complain and because of that we have not done anything because we cannot address anybody....You know most of the patients are afraid to come up because they are afraid we might treat them badly after" *(Female counsellor, 27 years old)

The participants stated that efforts have been made to address confidentiality within the treatment centres. They revealed that the hospital authorities regularly organise workshops and seminars for staffs where confidentiality is re-emphasized. Furthermore, some stated that only staffs directly involved in the management of patients have access to their medical records. Additionally, they mentioned that HIV results are documented with special codes as a protective measure from parties not directly concerned with the management of patients.

*"Now there is a strategy we have put in place that HIV result of patients is not known to every staff who works in the unit. Only the counsellor and nurses in charge of that patient, and the doctor.... When the patient is sent to the lab, the lab test request slips are carried by the counsellor and the result is written with codes so that not everybody can understand" *(Male counsellor, 33 years old)

### Dealing with partner notification

Encouraging HIV-positive patients to disclose their status, especially to their sexual partners was an important challenge faced by the participants. They mentioned that despite the improvement in their counselling skills due to the trainings received and from their work experience, they still faced difficulties convincing some patients to voluntarily inform their partners about their HIV status. The major reason cited was fear of marital problems which included blame, verbal or physical assault, and even divorce. Based on the participants' experiences, the refusal to notify the sexual partners was commoner amongst male patients who were more likely to have been promiscuous prior to their diagnosis. They further had experienced that patients who disclosed their status were more likely to engage in safer sex, had better treatment compliance, and outcomes compared to those who concealed their status.

*"I think from experience this is common with men and it is just because of their lifestyle. You know when they have so many women and finally when they become sick.... they will not want to tell their wives... so the ones revealing their status usually follow-up treatment very well. Some will not even use condoms with their wives because they don't want their wives to know they are HIV-positive" *(Female counsellor, 48 years old)

The four positions taken regarding partner notification are represented in the positional map (Figure 1). The positions include the following: *"Respecting patients' autonomy is the benchmark of counselling" *(position one); *"While safeguarding patients' autonomy, patients should be encouraged to disclose their status to their partners for family benefits" *(position two); *"Protecting patients' sexual partners at risk is essential but counsellors should be legally protected" *(position three); and *"HIV should be regarded as a chronic disease; testing and disclosure should be made routine" *(position four). It is noteworthy that, although the positions are presented figuratively and in a somewhat linear manner, the participants' perceptions were not static. Many participants shared multiple views simultaneously and these seemed to change over time depending on the legal and ethical obligations at their disposal. The dynamic nature in their views highlights the complexities surrounding partner notification for HIV. It is also critical to state that positions one and two reflect the current counselling practices in partner notification for HIV in the region/country, while positions three and four are the participants' wishes for future policies.

#### Position one

The reflections in this position focused on absolutely respecting patients' autonomy as enshrined in the counselling training and professional ethics. This position was shared mostly by the fairly younger counsellors with few years of work experience. The participants declared that during counselling, patients are provided with the basic information about HIV, the benefits of testing and disclosing their status to their relatives, and regarding informed consent. Therefore, if for whatever reasons patients object to inform their sexual partners about their HIV status, it was not the counsellor's duty to do otherwise without the patients' consent. According to them, endorsing that counsellors should disclose patients' results to their sexual partners constitute a violation of their professional ethics which could attract undesirable consequences.

*"No we cannot do that. That will be against our professional ethics because we are not supposed to disclose a patient's information without his consent. If we do that we might run into problems with the authorities *[the hospital administration] *because they will say we have breached confidentiality." *(Male counsellor, 33 years old)

This position was also shared by a legal expert who stated that although the existing national laws have not been revised to specifically protect people living with HIV/AIDS (PLWHA) against discrimination, health care providers who disclose HIV-positive patients' results to their sexual partners without their consent could be prosecuted on the grounds of breach of professional ethics.

*"In court that *[notifying an HIV-positive patient's partner] *can be argued in terms of breaching professional ethics because as you know ethically it is wrong to disclose your patient's result or diagnosis to third parties without that individual's consent. But to say in strict terms that there is an existing text with particular reference to maintaining confidentiality with regards to HIV-positive patients' results is a misnomer." *(Male judge, 55 years old)

#### Position two

This position incorporates both safeguarding patients' autonomy and their partners' safety which is beneficial to the entire family. This view was generally shared by the more experienced counsellors. Although the participants who held this view acknowledged the importance of respecting patients' autonomy, they felt that it was their duty to also protect patients' sexual partners at risk of HIV exposure, and to enable them to seek prompt treatment if already infected.

*"The law states that we should respect peoples' privacy or confidentiality...but I like to inform the partner because I know from experience that people who refuse to disclose to their partner will infect them....what if the wife comes tomorrow and is diagnosed positive?... It will be my fault because I did not inform her..." *(Male counsellor, 35 years old)

The participants emphasized that their training and work experience have improved their communication skills and relationship with the patients immensely. They remarked that if counselling is properly done and much time spent with the patients to gain their trust, the patients would see the need for testing and subsequently informing their partners about their HIV status.

*"When we started...most of us were inexperienced. We never knew how to present most of the things to most of the patients but with the trainings we take our time to give the best counselling to the patients so that they will not have misconceptions...it has made most of the patients to be understanding." *(Male counsellor, 35 years old)

In contrast to position one where the participants felt that nothing could be done if patients object to notify their partners, the participants in position two were ingenious at devising strategies to encourage and ensure that patients willingly notify their partners. These strategies included the following: i) couple counselling, although they stated that some men were reluctant to participate, ii) continuous or ongoing counselling of recalcitrant patients, educating them on the benefits of disclosure, iii) seeking consent from the patients to directly inform their partners in the patients' presence in scenarios where patients lacked the courage to do so personally, and iv) contact tracing, whereby the counsellors obtained telephone numbers or physical addresses of patients' partners and could directly inform them about the possibility of exposure to HIV without releasing the identity of the index patient. However, some participants who regularly used the last approach acknowledged that contacting faithful partners in relationships could result in adverse consequences since the partners would definitely know the source of the exposure.

*"One other thing that we have developed is contact tracing where those who are afraid to disclose to their partners we ask them to give us the telephone number of their sexual contact or contacts and we call the partner but we do not release their identity....The problem here is that if the partner has been faithful, she will definitely know that it is the husband who has infected her and it will cause problems in the house." *(Male counsellor, 41 years old)

#### Position three

The focus in this position was on protecting the sexual partners at risk of HIV, and providing prompt treatment to those already infected since this will be beneficial to the entire family in the long run. This position, most often was shared by participants from the faith-based centres irrespective of their age and experience who incorporated morality issues when encouraging HIV status disclosure. They opined that upholding confidentiality in absolute terms was morally wrong, and patients who refused to inform their partners about their status were selfish by not considering the health and wellbeing of their partners. Although in favour of counsellors to directly notify the sexual partners of such uncooperative patients, the participants stated that they could not do otherwise because of the legal constraints.

To back this position, some participants stated that after all attempts to encourage voluntary disclosure failed; they occasionally 'threatened' their patients to make them notify their partners. Although they eventually respected the patients' autonomy, they claimed this measure was only used as a last resort to encourage voluntary disclosure.

*".... if I try other measures and don't succeed, I will tell you that if you don't do it I will do it for you. When you do that many will not want you to be the one to do it. They will rather prefer to do it themselves....it is just a way of getting around because if they resist we will not do it." *(Female counsellor, 48 years old)

The participants mentioned that in scenarios where patients refused voluntary disclosure, they were constantly in a dilemma between respecting patients' confidentiality and disclosing the status to their sexual partners. This was even more disturbing if they were acquainted with the patients' sexual partners

*"I think it is different if you know the man is positive but you have never met the wife. In this case *[the counsellor is acquainted with the wife] *the woman came to you. What will you tell? A lie and then she will not trust you because she will discover it later on and it will be worse.... personally I will not sleep well." *(Female counsellor, 33 years old)

However, they all acknowledged that in order for them to notify noncompliant patients' sexual partners, the government has to step in with a policy that legally protects health care providers against litigation.

*"My proposal is that there should be a law or by law protecting the health professionals in such special cases that they have the right to protect partners for public health benefits." *(Male counsellor, 27 years old)

A legal practitioner partly endorsed this position stating that sexual partners in a legal relationship deserve to know the HIV status of their partners. He therefore did not regard direct disclosure of HIV-positive patients' results by counsellors to their legal partners as a violation of the patients' autonomy.

*"....It is your basic human rights for your results not to be disclosed to a third party but at the same time it also my basic human rights that I should know what you are sick of if you are my partner so that I can take care of myself if it warrants so. Therefore for you to refuse to disclose your status to me is a violation of my own human rights. There is a limit to privacy especially in a marital context because I see no violation in a partner's privacy if he or she is HIV-positive but refuses to disclose his result to the other partner and a health official does so to protect that partner and the entire family." *(Male lawyer, 47 years old)

Notwithstanding, some participants acknowledged that endorsing partner notification by health care providers without the patients' consent could deter patients from seeking treatment. They further stated that it could lead to marital disharmony including divorce for which the counsellors would ultimately be blamed for by the concerned parties and society.

#### Position four

In position four, the emphasis was on addressing HIV/AIDS as any other chronic and treatable disease. This view was most commonly shared by male counsellors from faith-based hospitals. They mentioned that during the pre-ART era, recommending testing for HIV without offering treatment deterred testing. Following the scale-up of free life-time ART to all eligible persons in the country, there is the need to make HIV testing and disclosure normal and routine processes. According to these proponents, this measure will benefit many by prolonging peoples' lives and making them more productive in society. Although they acknowledged that such a policy would be difficult to implement, they suggested that it was an initiative worth considering in the not too distant future.

*".....because the drugs are available now for free, I don't see why we should not consider it *[routine testing and disclosure of HIV result] *as in every other diseases like diabetes, hypertension where people come for check up every time and they also have to take their drugs for life." *(Male counsellor, 33 years old)

## Discussion

The respect for patients' confidentiality is a fundamental principle in medical ethics, and also a legal duty that health care providers owe to their patients. However, the respect of absolute confidentiality has been a subject of debate [24-26]. With the advent of the HIV/AIDS epidemic, this debate has been re-echoed if confidentiality should be compromised when HIV-positive patients refuse to voluntarily notify those at risk of infection, especially their sexual partners [27-29]. Disclosing one's HIV status is particularly difficult because HIV is often associated with sexuality, coupled with the double stigma and discrimination experienced by patients co-infected with TB and HIV [20,30]. Our study revealed that counsellors experienced the dilemma between upholding their professional ethics by not disclosing their patients' HIV status to their sexual partners, and being morally upright by doing so when confronted with patients who refused voluntary disclosure. This therefore indicates the need to visualise the complexity of partner notification in order to move forward.

Our study demonstrated that upholding confidentiality and respecting patients' autonomy is emphasized during the counsellors' training and practice in all the study settings. However, the absence of a professional code of ethics within some centres is a call for concern since it is important for counsellors to know the existing laws regarding confidentiality and their professional ethics. Moreover, to foster a lasting patient-provider alliance and maintain patients' trust in the health care system, it is inevitable that patients are properly educated on their rights to confidentiality and autonomy.

Partner notification for HIV is generally regarded as an ethical and legal issue, and position one underscored the importance of respecting patients' confidentiality and autonomy. This position is drawn from the personal autonomy framework that is much more linked to biomedicine. Participants who held this opinion were mostly young and relatively inexperienced, and this could be understood from their apparent lack of adequate counselling skills. This might have limited their negotiating power to encourage patients to voluntarily inform their partners about their HIV status. They however provided justification for upholding this position which had both legal and ethical dimensions since they asserted that a counsellor's duty to the patient supersedes every other duty. Consequently, patients should not be forced to disclose such information for the benefit of others. This argument is consistent with Kantian theory which postulates that human beings deserve to be treated with respect as ends in themselves and not as means to another individual's ends [31].

The dilemma in public health between respecting individual rights in order to foster a trusting patient-provider relationship versus protecting the collective rights of the sexual partners was highlighted in position two. Resolving such conflicts was a daunting task to the counsellors since it entailed acting within the limits of the law, their professional ethics and morality. The salient difference between the participants in this position and those in position one lies in the fact that they were 'morally autonomous'. This implies that their decisions were based on moral principles and understanding of the situational facts from which they acted upon their considered judgment [32]. This is expected since the participants who held this position were mostly older and more experienced counsellors. Although they respected the law and professional ethics regarding confidentiality, they did not literally pursue these rules without appreciating the rationale for applying them. This was reflected in the various strategies which they devised to encourage uncompromising patients to voluntarily notify their sexual partners about their HIV status. This position regarding counselling and disclosure of HIV status seems to be the dominant position, and it is the policy recommended by UNAIDS and WHO [3,6]. This policy incorporates the human rights framework into public health as a response to the HIV/AIDS epidemic. Human rights is a reflection of deeply held values of what states and governments should not do and what they should ensure to all its citizens [33]. The UNAIDS/WHO policy therefore promotes and protects public health, and also ensures that the human rights and dignity of HIV-positive people are not violated. In developing countries where the brunt of the HIV epidemic exists, patient referral has been found to be the most preferred method in partner notification strategies [34]. However it does not address the situation whereby patients deliberately conceal their status from their partners thereby placing them at risk despite counsellors' efforts to encourage voluntary disclosure.

Position three is a wish to address the above concern since participants who held this stance advocated for conditional confidentiality and legal protection to notify patients' sexual partners. This position received legal backing with the premise that it is a violation of the sexual partners' human rights if health care providers refuse to inform them about their diagnosed partners' HIV status. However, this should be done for beneficial reasons in cases where such patients had refused to voluntarily do so. The legal backing was nonetheless restrictive in the sense that conditional referral advocated in such a scenario was limited only to partners in legal relationships. This implied that no protection would be available for unmarried partners which could eventually potentiate HIV transmission in the society. However, the assertions in this position are consistent with the argument that the right to confidentiality is only possible between morally sensitive people. Therefore, individuals who place themselves 'beyond the pale', implying that their actions are potentially harmful to others forfeit the right to confidentiality [29]. The conditional referral strategy advocated for in this position is therefore grounded on utilitarianism which asserts that 'decisions should be judged by their consequences, in particular by their effect on the total sum of individual wellbeing' [31]. Our study also revealed that the participants were sometimes frustrated when patients objected to voluntary partner notification especially if the participants were loyal to the sexual partners. There is a need for proper education and sensitisation of counsellors, and the society in general about sexual ethics. This is the ethics within partnerships of mutual respect, consent, and shared responsibilities for sexual behaviour and its social, emotional and health consequences [10] that are fundamental to human rights. Moreover, the creation and enforcement of an official AIDS law that protects both the rights of PLWHA especially against stigma and discrimination, and that of their partners would facilitate voluntary disclosure. In addition, the existing laws and customs which discriminate against women, favours male dominance, polygamy, adultery, and fails to criminalise domestic violence including rape against women needs to be revised [35]. This is important as participants stated that patients were apprehensive of disclosure since it could attract negative consequences in relationships including divorce which have been reported in other studies [1,13]. Furthermore, partner notification protocols should be made available, and the counsellors properly educated that the duty to protect partners at risk should be based on ethical and legal justifications and not because of personal sentiments and loyalty to a particular partner [32]. Most importantly, the concept of 'proportionality' [36] should be applied in cases of provider referral. This implies that in scenarios where public health ethics confronts individual human rights, public interests subvert individual rights but there should be absolutely minimal infringement on the individual rights. These measures will help to address some of the complexities in HIV counselling and facilitate voluntary partner notification.

Besides the fact that conditional referral could exacerbate the negative consequences of voluntary disclosure, it was highlighted in this study that enforcing conditional referral could also deter prospective patients from seeking treatment. This is consistent with the views of the proponents of unconditional confidentiality [24,25]. This is a worthy concern especially in developing countries where alternative medicine is an integral part of the health care system [37], and could attract such disgruntled patients towards these services. Despite these arguments, there is lack of empirical evidence in support of the undesirable consequences of implementing provider referral approach in partner notification. Provider referral has been documented to be effective in identifying sexual partners at risk in developed countries [7,38,39], and is generally permitted as a supplement to patient referral in North America [40] and Europe [41,42]. However, its feasibility and effectiveness in developing countries with different socio-cultural and political contexts is limited [43] and requires further evaluation.

The perception held in position four was that HIV should be regarded as any normal medical infection, whose diagnosis and disclosure should be based on medical as opposed to ethical or legal necessities. The implication of this approach is that HIV testing and partner notification could move from the counsellors to the physicians and thereby making the counsellors redundant. Alternatively, this position could normalise HIV/AIDS into a medical condition just as any other. The latter alternative was the premise for the participants' argument in support of routine HIV status disclosure since life-saving ARTs are now provided free of charge to all eligible persons in the country. Applying this approach would ultimately be beneficial both to the patients and those at risk since it would reduce HIV transmission, and increase patients' access to HIV services. It has been argued that 'HIV exceptionalism', whereby public health response in the early years of the HIV epidemic had been fundamentally different from other sexually transmitted infections and public health threats, has been the reason for the lag in global HIV prevention and control [44]. It is further argued that 'exceptionalism' has enhanced the stigma associated with HIV infection and led to the confusion between secrecy and confidentiality, and consequently promoting the silence around HIV/AIDS [45]. These arguments have prompted the advocacy for routine HIV testing [45], and third party disclosure [46] as a norm in health care settings in order to demystify HIV infection as highlighted in position four. The justification being that 'normalization' of HIV/AIDS is not a threat to individual human rights but rather failure to prevent HIV infection is an infringement on human rights' [45].

### Methodological considerations

Using a positional map in this study was very useful in delineating the full spectrum of the reflections regarding partner notification for HIV within and across groups of counsellors. This enabled us to visualise positions that were taken in the data which ultimately facilitated the formulation of ideas regarding the implications of these positions in counselling practice, and in HIV prevention and control. However, there are limitations in our study that deserve consideration. First of all, our study sites were purposively selected and therefore the participants' perceptions do not necessarily reflect those of the entire counsellors in the region. Notwithstanding, since our study sites are approved TB/HIV treatment centres which also serve as referral centres in the region, we believe they share similar characteristics and challenges to the other smaller centres. Additionally, since the first author was acquainted with the participants, either they may have provided less information assuming he was already familiar with the settings or they could have provided favourable responses to demonstrate their achievements. Although these limitations might affect the extent to which our results could be generalised within the region and to similar contexts with generalised HIV epidemics, we believe our findings have provided valuable insights into the complexities faced by counsellors in the process of encouraging voluntary partner notification of HIV status and how these could affect policy.

## Conclusions

The ethical, legal and moral dilemmas between respecting individual patient's rights and the collective rights of patients' sexual partners encountered by counsellors were highlighted in this study. Although confidentiality is important in the health care provider-patient relationship, there are exceptions where this can be subverted. However, this should be balanced to ensure that the interests of both the patients and their sexual partners are served. This process will be facilitated by adopting a human rights framework which recognises both individual and collective rights. Following the scale-up and universal access to HIV services, there is a need to re-examine partner notification policies and taking into consideration the contextual factors which might affect the feasibility and acceptability of a particular approach (or a combination of approaches). This will ensure that HIV transmission is significantly curtailed, and those at risk identified in time and provided with the necessary HIV services. Proper counselling guidelines addressing the legal and ethical challenges in HIV/AIDS should be made available to counsellors and within the treatment centres to facilitate the counselling process. Meanwhile, approaches like contact tracing, counsellor-mediated patient referral for patients who lack the communication skills to disclose to their partners should be considered. In addition, continuous counselling for inflexible patients particularly on shared responsibility in relationships, and couple counselling where partners are encouraged to mutually disclose their status should be thoroughly explored and expanded where appropriate.

## Competing interests

The authors declare that they have no competing interests.

## Authors' contributions

BNN and AKH conceptualized and designed the study; BNN performed data collection, analysed and drafted the manuscript. All co-authors interpreted the results and provided substantial revisions of the manuscript. All the authors have read and approved the final version of the manuscript for submission.

## Pre-publication history

The pre-publication history for this paper can be accessed here:

http://www.biomedcentral.com/1472-698X/11/6/prepub
